# Trans-Synaptic Spread of Amyloid-*β* in Alzheimer's Disease: Paths to *β*-Amyloidosis

**DOI:** 10.1155/2017/5281829

**Published:** 2017-12-24

**Authors:** Annabella Pignataro, Silvia Middei

**Affiliations:** ^1^Department of Experimental Neurology, Laboratory of Psychobiology, Santa Lucia Foundation, Via del Fosso di Fiorano, 00143 Rome, Italy; ^2^Institute of Cell Biology and Neurobiology, National Research Centre, Via E. Ramarini, Monterotondo, 00040 Rome, Italy

## Abstract

Neuronal activity has a strong causal role in the production and release of the neurotoxic *β*-amyloid peptide (A*β*). Because of this close link, gradual accumulation of A*β* into amyloid plaques has been reported in brain areas with intense neuronal activity, including cortical regions that display elevated activation at resting state. However, the link between A*β* and activity is not always linear and recent studies report exceptions to the view of “more activity, more plaques.” Here, we review the literature about the activity-dependent production of A*β* in both human cases and AD models and focus on the evidences that brain regions with elevated convergence of synaptic connections (herein referred to as brain nodes) are particularly vulnerable to A*β* accumulation. Next, we will examine data supporting the hypothesis that, since A*β* is released from synaptic terminals, *β*-amyloidosis can spread in AD brain by advancing through synaptically connected regions, which makes brain nodes vulnerable to A*β* accumulation. Finally, we consider possible mechanisms that account for *β*-amyloidosis progression through synaptically linked regions.

## 1. Introduction

Gradual accumulation of *β*-amyloid peptide (A*β*), the proteolitic product of amyloid precursor protein (APP), is the principal cause of synaptic dysfunction and cognitive loss in Alzheimer disease [1]. The events leading to amyloidogenic APP processing have been a subject of intense research, with implications for understanding regional vulnerability of specific brain regions to progressive A*β* accumulation (see glossary in Table 1 for a definition of the different aggregation states).

APP is a type-I transmembrane protein highly expressed in neurons, where it regulates synaptic function and neurite outgrowth [2–4]. Two alternative enzymatic pathways process APP: in the nonamyloidogenic pathway, APP is subjected to consecutive cleavage steps through *α*- and *γ*-secretases that cut APP within the A*β* fragment. In the amyloidogenic APP processing, *β*-secretases cut the A*β* fragment at its beginning and the consecutive *γ*-secretase cleavage releases A*β* peptides (see [5] for an exhaustive review of APP processing).

Neuronal activity directly regulates APP processing. Upon neuronal activation, APP is trafficked into endocytic compartments, where it is processed [6, 7]. In neurons that overexpress APP, activity favours *β*-secretase-mediated APP processing [8] and lowers the endocytosis of the *α*-secretase enzyme ADAM10 [9]. In line with this, in AD brains, amyloid aggregates in regions with intense neuronal activity, like the ones with baseline high levels of activity, making those regions particularly prone to AD degeneration.

However, several studies have reported some exceptions to the activity A*β* relation. On one side, baseline active regions become hypoactive once A*β* has accumulated and start to damage neurons, and stratifications between pre- and postamyloid stages must be taken into account when considering the impact of activity on AD progression [10]. For instance, longitudinal studies indicate that A*β* accumulation can start 10–15 years before the occurrence of cognitive symptoms and that regions which are more active and accumulate more A*β* are the ones that later degenerate and then become hypoactive in concomitance with cognitive loss [10, 11]. On the other side, not all regions that display high baseline activity are subject to A*β* accumulation, suggesting that neuronal activity is not the only player in regional vulnerability.

In the present review, we propose a critical analysis of possible mechanisms involved in regional accumulation of A*β* and try to extend the understanding of the complex relationship between neuronal activity and AD progression.

First, we report studies that found A*β* accumulation in specific brain areas and we focus on the evidence that regions with elevated neuronal activity due to the convergence of several synaptic pathways (brain nodes) are more vulnerable to A*β* accumulation. Next, we will examine data in support to the idea that, since A*β* spreads through synaptically connected regions, the elevated concentration of synapses makes brain nodes vulnerable to A*β* accumulation. Lastly, we consider some possible mechanism to explain propagation and gradual accumulation of A*β*, which received little attention so far.

## 2. Nonlinear Link between Neuronal Activity and A*β* Accumulation

One hypothesis that has emerged over the past decade suggests that intense neuronal activity makes brain regions more vulnerable to A*β* accumulation. This hypothesis originates from evidences reporting that regions with elevated basal activity are more prone to accumulate A*β* aggregates [12] and is supported by studies demonstrating a causal role of neuronal activity in A*β* generation and secretion [8, 13]. In this context, two pivotal studies have reported that the amount of small monomeric A*β* species rises in the interstitial brain fluid (ISF) while awake or in conditions of active neurological status but decreases during sleep [14, 15], thereby providing strong physiological proof to the idea that neuronal activity can dynamically regulate extracellular A*β* in vivo.

### 2.1. Experimental Modulation of Neuronal Activity

The idea of inactivity-vulnerability link received further support with studies that have modulated neuronal activity in specific brain regions of living AD mice.

A few studies investigated the effects of whisker manipulation on A*β* levels in the barrel cortex, a neuronal circuit that receives physiological inputs from the vibrissae of mice [12, 16, 17]. In AD Tg2576 mice, 30 minutes of mechanical vibrissae stimulation, which associates with rises of neuronal activity in the barrel cortex, was sufficient to increase the amount of ISF A*β* in this region [12]. On the contrary, acute vibrissae trimming that inhibited neuronal activity of the barrel cortex reduced extracellular A*β* (and thereby amyloid plaques) in the same cortical region [12, 16], an effect that was likely due to a significant rise of intracellular A*β* [16, 17].

More recently, one study on A7 AD mice [18] used optogenetics to stimulate the presynaptic fibers of the perforant pathway (PP) that links the entorhinal cortex (EC) and the hippocampal dentate gyrus (DG), a region particularly prone to A*β* accumulation. ISF A*β* levels were increased more than 20% in the hippocampus after short pulses of PP light stimulation. Furthermore, postmortem immunohistochemistry revealed that repeated optogenetic stimulations over a 5-month period significantly increased the A*β* burden in the hippocampal DG.

On the same experimental train of thought, another study [19] used a chemogenetic approach [20] to modulate neuronal activity in the posterior parietal association and posterior somatosensory cortex of aged 5xFAD and PS/APP mice. In this study, chronic chemogenetic activation led to a significant increase in thioflavin-positive plaques and chronic chemogenetic inhibition reduced plaques and the surrounding oligomeric A*β* species in activated and silenced regions, respectively.

### 2.2. Default Mode Network and Brain Hubs

In humans, one important branch of studies on the impact of neuronal activity on A*β* levels has focused on the default mode network (DMN), a set of interconnected brain regions that are most active at resting state. Buckner and collaborators used functional magnetic resonance imaging (fMRI) to identify patterns of network activity and positron emission tomography (PET) with PIB compound to visualize amyloid deposits in elderly patients. Their studies reported spatial overlap between the topography of amyloid deposition and the regions of DMN (including posterior cingulate and parietal cortex, medial temporal lobe, and medial frontal subsystem) [21, 22]. Using aerobic glycolysis, a parameter that is based on the excess of glucose utilization as a measure of basal metabolic activity, other researchers [23, 24] found a strong correlation between baseline metabolic activity in DMN regions and A*β* deposition. Also, Sperling et al. [11, 25] found that in at-risk subjects that are still clinically intact, aberrant DMN activity is predictive of amyloid deposition, thereby providing support to the corollary idea that hyperactivity is a “prime” event in A*β* accumulation. We will come back to hyperactivity later in this paper.

However, hypoactivity has been also reported in some of DMN regions in healthy subjects carriers for APOE*ε*, an important risk factor for the development of late-onset AD [26] (see also [27]). APOE is a lipoprotein involved in cholesterol removal, but in the brain, it is involved in development and plasticity mechanisms. Of note, APOE interacts with A*β* and accelerates the processing of amyloid deposition [28]. As mentioned above, hypoactivation can be found in DMN regions upon amyloid deposition, and it is possible that hypoactivation reported in APOE*ε* carriers depends on advanced amyloid deposition.

Together, the above studies highlight strong correlations between neuronal activity of DMN and A*β* deposition, but the relationship between the two is not always linear. One longitudinal study [29] that combined fluoreodeoxiglucose (FDG) for metabolic measure and Pittsburgh compound B to detect A*β* deposits found that regions of high metabolic consumption in young adults overlap with regions of fibrils later in life, but this linear relation was not detectable in some of the brain regions that were included in the study. For instance, increased amyloid burden in the anterior cingulate cortex, medial frontal cortex, and lateral temporal cortex was not associated with a high metabolic rate during the lifespan. Conversely, visual cortex was spared by A*β* accumulation despite its high metabolic activity.

One important clue to understanding this apparent paradox comes from studies on brain hubs, represented by nodes with an elevated number of connections, largely overlapping with heteromodal association cortices included in the DMN. Buckner and coworkers [30] run a complex computational analysis with the goal to extend their previous DMN studies to research on hubs [21, 22]. The authors found that cortical regions with elevated interconnectivity (including posterior cingulate, lateral temporal, lateral parietal, and medial/lateral prefrontal cortices) display elevated A*β* deposition. Interestingly, the study also showed that primary sensory areas have relatively little connectivity, which could explain the reason for low A*β* depositions in the visual cortex despite intense activity in this region [29].

Hence, strong network connectivity rather than elevated baseline activation can be at the origin of vulnerability to A*β* accumulation in active brain regions. In a study that directly explored this possibility through stepwise connectivity method in PIB-PET images, Sepulcre et al. [31] found connectivity links between regions that display elevated amyloid-*β* accumulation. In particular, strong links were evident between the hippocampal region and orbitofrontal, lateral temporal, and posterior cingulate cortex.

Bero et al. replicated these results in an AD mouse model [32]. By mapping functional connectivity through optic intrinsic signal (fcOIS) in the brain of young APP/PS1 transgenic mice, the authors found that some regions display elevated interconnectivity, and the magnitude of this interconnectivity is predictive of plaque deposition at successive age points [32].

## 3. *β*-Amyloidosis Spreads through Synaptically Connected Regions

Since synapses are the principal sites of A*β* release [8, 13], one possible explanation of why A*β* accumulates in brain nodes is that A*β* peptides released from synaptic terminals gradually accumulate in the extracellular space of downstream regions.

This prediction has been confirmed at the synaptic circuits between EC and the DG. Sheng et al. [33] found that APP processed in the EC neurons is the major A*β* source on the hippocampus. In this study, APP Swedish/preselinin1 mice subjected to unilateral ablation of EC had 45% reduction in thioflavin-positive A*β* deposits in hippocampal DG of the lesioned hemisphere with respect to the control (nonlesioned) one. Since APP is axonally transported from EC neurons to nerve terminals in the DG through PP projections [34–36], Sisodia and collaborators [37] asked if blocking this access pathway can prevent A*β* deposits in the DG. Using transgenic mice that overexpress FAD-linked mutant APP and PS1, the authors demonstrated that transection of PP is sufficient to decrease amyloid burden 2-3-fold in the hippocampus (and up to 5.5–10-fold in the DG) with respect to nonlesioned controls.

A more direct evidence of spreading from EC to DG came from Mucke's group [38]. These authors crossed Tet-APP mice with NOP-tTA mice, in order to generate chimeric EC-APP mice with expression of APP with humanized A*β* domain restricted to the II and III layers of the EC. A*β* deposits, which were initially present only in the EC of these mice, became progressively evident in the DG, thereby leading the authors to argue that A*β* spread from the EC to the hippocampus. Even though the following paper discussed the validity of the APP-EC mouse model [39], Harris' study [38] was confirmed by other researches, which also expanded their results to different brain regions. Among these, a set of experimental framework generated a transgenic mouse model of AD expressing single Arctic APP mutation selectively in the subiculum (TgAPParc) [40, 41]. In these mice, A*β* deposition was found to start from the subiculum and then spread in a time-dependent manner to synaptically connected brain regions (including the thalamus retrosplenial cortex, mammillary bodies, fimbria, fornix, motor cortex, and sensory cortex), and this spreading pattern was abolished by ablation of the subiculum [42].

Collectively, the above data indicate that A*β* is preferentially released at brain nodes and those connections between nodes represent preferential routes for the spreading of *β*-amyloidosis.

## 4. Possible Mechanisms of Spread

The passage of disease-associated misfolded proteins from neuron to neuron is a key mechanism of progress in neurodegenerative diseases. Although the mechanisms of spreading have been clarified for some neurodegenerative diseases (including *α*-synuclein in Parkinson and Tau in AD, see [43] for an exhaustive review of spread hypothesis), not much is known about the spread of the *β*-amyloid protein. In this last section, we provide a short review of possible mechanisms involved in A*β* spread, which have been investigated over the last few years.

### 4.1. Prion-Like Propagation

Administration of A*β*-containing extracts can accelerate A*β* aggregation in the brain of living animals [44–46]. This piece of evidence led researchers to question if small peptidic A*β* “seeds” which are released from synaptic terminals in one brain region can trigger A*β* accumulation in other regions.

One set of studies directly investigated this possibility [47–49] by performing biominulescence imaging on APP23 gfap-luc mice unilaterally inoculated with purified A*β* aggregates. In these mice, widespread bilateral A*β* signal was found through the whole brain, supporting the possibility that A*β* seeds contribute to the spreading of amyloidosis [47].

In another study, A*β*-containing brain extracts from aged APP23 mice were injected into distinct brain regions (including olfactory bulb, parietal cortex, striatum, and hippocampus) of young and still plaque-free mice of the same transgenic strain [46]. Three months later, A*β* deposition was found in proximity of some of the injected brain regions (including the entorhinal cortex and hippocampus) and six months later A*β* deposits resembling amyloid plaques were also found in adjacent (and otherwise plaque-free) brain regions, indicating that Ab spreads from the injection site and gradually accumulates in other sites.

This evidence raises the question of whether seeds trigger an infectious process that propagates within interconnected regions or rather if the seeds accelerate a process that would spontaneously occur in brain tissues that are prone to *β*-amyloidosis. A set of experimental evidences excludes the possibility of an infectious process: (i) when other routes of A*β* extract administration are used (including intranasal, intraocular, or oral) A*β* deposits are not evident in downstream regions [46] as would be expected in case of an infectious transmission; (ii) A*β* depositions have not been found in mice expressing human APP wild-type, which is not prone to *β*-amyloidosis [44] (see also [49]). Similarly, (iii) A*β* does not accumulate in brain regions that typically do not form plaques, for instance, cortical regions that receive projections from the striatum [46].

Collectively, the above evidence indicates that although A*β* seeds can spread from one brain region to one other, likely passing through synaptic connections, this step is not sufficient to form deposits. Rather, A*β* only accumulates when seeds reach “receiving” regions that are prone to amyloidosis.

### 4.2. A*β* Generation from Postsynaptic Neurons

The above studies suggest that, even if the convergence of several synaptic inputs makes brain regions particularly vulnerable, factors associated with intrinsic properties of receiving regions are likely participating in the spreading of amyloidosis. No studies so far directly investigated this possibility, which is rather supported by indirect evidences showing that A*β* released into the extracellular space alters the physiological activity of downstream neurons. For instance, A*β* causes reduction of synaptic glutamate reuptake [50] and/or enhanced glutamate release at presynaptic terminals [51] that act together to increase extracellular glutamate level and promote hyperactivity. Aberrant neuronal activity has been viewed as a beneficial compensatory effect aimed at recruiting neuronal resources, but because of its strong positive correlation with APP processing, it could rather participate to a positive feedback loop that triggers further APP cleavage from hyperactive neurons and cause A*β* release from their dendrites [52, 53].

Hence, one possible scenario, which is worthy of future investigation, is that hyperactivity spreads from one brain region to synaptically connected one(s), thereby favouring activity-dependent A*β* release from downstream neurons.

## 5. Conclusions and Future Prospectives

We here provide one hypothetic model to explain the vulnerability of selected brain regions to A*β* accumulation. We suggest that most of the vulnerable regions in AD brain are nodes that receive high convergence of synaptic connections. One simple explanation for this vulnerability is that synaptic circuits are preferential routes for the release of A*β*, which then progressively accumulates at postsynaptic sites. But other factors intrinsic to neurons within the brain nodes can also contribute to *β*-amyloidogenesis through the spread of hyperactivity.

This model raises questions which should be addressed in future studies. For instance, if the convergence of synaptic inputs makes brain nodes vulnerable, can we reduce the amount of synaptic inputs without interfering with general cognition? As a first step, this question could be addressed in AD mouse models using the abovementioned optogenetic strategies that allow to silence specific synaptic inputs in selected brain regions of behaving mice.

Systemic administration of antiepileptic drugs can improve memory performance in patients at early AD stages [54], but with relevant side effects. Hence, it could be therapeutically relevant to investigate if the delivery of these drugs can be restricted to brain nodes in order to potentiate their silencing power and to limit undesired effects.

## Figures and Tables

**Table 1:** A*β* type and aggregation state.

A*β* type/aggregation state	Detection method	References
A*β* oligomers	ELISA	[8, 13–17, 48, 52] [12, 19, 32]^∗^ [18, 38, 40, 41, 47]^∗^# [37]#
	Western blot	[16, 17, 48] [44]^∗^ [46]^∗^# [47]#

A*β* protofibril	Immunoistochemistry	[19]^∗^

A*β* plaques	Immunoistochemistry	[12, 19, 44, 45]^∗^ [16, 17, 33, 48] [16, 37, 38, 40–42, 46, 47]^∗^#

Amyloid deposition in human brain	Pittsburgh compound B (PIB) PET	[11, 25, 29]^∗^ [19–22, 31]

*Amyloid-β (Aβ)* refers to peptides derived from cleavage of the amyloid precursor protein (APP) by *β*- and *γ*-secretase. *Soluble Aβ oligomers* indicate A*β* species formed by the aggregation of more than one A*β* peptide (monomers). Oligomers are commonly detectable in sample solutions following centrifugation. *Aβ protofibrils* are aggregates of A*β* oligomers, with lengths of over 40 nm. *Aβ fibrils* are A*β* aggregates composed predominantly of *β*-sheet structure and resistant to degradation. These aggregates are often found in proximity of amyloid plaques. *Amyloid plaques* consist in deposits of insoluble A*β*; ∗ indicates studies that report progressive of A*β* accumulation; # indicates studies that demonstrate A*β* spread between distinct brain regions.

## References

[1] Selkoe D. J. (2002). Alzheimer’s disease is a synaptic failure. *Science*.

[2] Leyssen M., Ayaz D., Hébert S. S., Reeve S., De Strooper B., Hassan B. A. (2005). Amyloid precursor protein promotes post‐developmental neurite arborization in the *Drosophila* brain. *The EMBO Journal*.

[3] Muller U. C., Zheng H. (2012). Physiological functions of APP family proteins. *Cold Spring Harbor Perspectives in Medicine*.

[4] Young-Pearse T. L., Chen A. C., Chang R., Marquez C., Selkoe D. J. (2008). Secreted APP regulates the function of full-length APP in neurite outgrowth through interaction with integrin beta1. *Neural Development*.

[5] Haass C., Kaether C., Thinakaran G., Sisodia S. (2012). Trafficking and proteolytic processing of APP. *Cold Spring Harbor Perspectives in Medicine*.

[6] Das U., Scott D. A., Ganguly A., Koo E. H., Tang Y., Roy S. (2013). Activity-induced convergence of APP and BACE-1 in acidic microdomains via an endocytosis-dependent pathway. *Neuron*.

[7] Das U., Wang L., Ganguly A. (2016). Visualizing APP and BACE-1 approximation in neurons yields insight into the amyloidogenic pathway. *Nature Neuroscience*.

[8] Kamenetz F., Tomita T., Hsieh H. (2003). APP processing and synaptic function. *Neuron*.

[9] Marcello E., Saraceno C., Musardo S. (2013). Endocytosis of synaptic ADAM10 in neuronal plasticity and Alzheimer’s disease. *The Journal of Clinical Investigation*.

[10] Jagust W. J., Mormino E. C. (2011). Lifespan brain activity, β-amyloid, and Alzheimer’s disease. *Trends in Cognitive Sciences*.

[11] Sperling R. A., LaViolette P. S., O'Keefe K. (2009). Amyloid deposition is associated with impaired default network function in older persons without dementia. *Neuron*.

[12] Bero A. W., Yan P., Roh J. H. (2011). Neuronal activity regulates the regional vulnerability to amyloid-β deposition. *Nature Neuroscience*.

[13] Cirrito J. R., Yamada K. A., Finn M. B. (2005). Synaptic activity regulates interstitial fluid amyloid-β levels in vivo. *Neuron*.

[14] Brody D. L., Magnoni S., Schwetye K. E. (2008). Amyloid-β dynamics correlate with neurological status in the injured human brain. *Science*.

[15] Kang J. E., Lim M. M., Bateman R. J. (2009). Amyloid-β dynamics are regulated by orexin and the sleep-wake cycle. *Science*.

[16] Tampellini D., Rahman N., Gallo E. F. (2009). Synaptic activity reduces intraneuronal Aβ, promotes APP transport to synapses, and protects against Aβ-related synaptic alterations. *Journal of Neuroscience*.

[17] Tampellini D., Capetillo-Zarate E., Dumont M. (2010). Effects of synaptic modulation on β-amyloid, synaptophysin, and memory performance in Alzheimer’s disease transgenic mice. *Journal of Neuroscience*.

[18] Yamamoto K., Tanei Z.-I., Hashimoto T. (2015). Chronic optogenetic activation augments Aβ pathology in a mouse model of Alzheimer disease. *Cell Reports*.

[19] Yuan P., Grutzendler J. (2016). Attenuation of β-amyloid deposition and neurotoxicity by chemogenetic modulation of neural activity. *Journal of Neuroscience*.

[20] Armbruster B. N., Li X., Pausch M. H., Herlitze S., Roth B. L. (2007). Evolving the lock to fit the key to create a family of G protein-coupled receptors potently activated by an inert ligand. *Proceedings of the National Academy of Sciences of the United States of America*.

[21] Buckner R. L. (2005). Molecular, structural, and functional characterization of Alzheimer’s disease: evidence for a relationship between default activity, amyloid, and memory. *Journal of Neuroscience*.

[22] Buckner R. L., Andrews-Hanna J. R., Schacter D. L. (2008). The brain’s default network: anatomy, function, and relevance to disease. *Annals of the New York Academy of Sciences*.

[23] Vlassenko A. G., Vaishnavi S. N., Couture L. (2010). Spatial correlation between brain aerobic glycolysis and amyloid-β (Aβ) deposition. *Proceedings of the National Academy of Sciences of the United States of America*.

[24] Vaishnavi S. N., Vlassenko A. G., Rundle M. M., Snyder A. Z., Mintun M. A., Raichle M. E. (2010). Regional aerobic glycolysis in the human brain. *Proceedings of the National Academy of Sciences of the United States of America*.

[25] Huijbers W., Mormino E. C., Wigman S. E. (2014). Amyloid deposition is linked to aberrant entorhinal activity among cognitively normal older adults. *Journal of Neuroscience*.

[26] Reiman E. M., Chen K., Alexander G. E. (2005). Correlations between apolipoprotein E ε4 gene dose and brain-imaging measurements of regional hypometabolism. *Proceedings of the National Academy of Sciences of the United States of America*.

[27] Filippini N., MacIntosh B. J., Hough M. G. (2009). Distinct patterns of brain activity in young carriers of the *APOE*-ε4 allele. *Proceedings of the National Academy of Sciences of the United States of America*.

[28] Kanekiyo T., Xu H., Bu G. (2014). ApoE and Aβ in Alzheimer’s disease: accidental encounters or partners?. *Neuron*.

[29] Oh H., Madison C., Baker S., Rabinovici G., Jagust W. (2016). Dynamic relationships between age, amyloid-β deposition, and glucose metabolism link to the regional vulnerability to Alzheimer’s disease. *Brain*.

[30] Buckner R. L., Sepulcre J., Talukdar T. (2009). Cortical hubs revealed by intrinsic functional connectivity: mapping, assessment of stability, and relation to Alzheimer’s disease. *Journal of Neuroscience*.

[31] Sepulcre J., Sabuncu M. R., Becker A., Sperling R., Johnson K. A. (2013). *In vivo* characterization of the early states of the amyloid-beta network. *Brain*.

[32] Bero A. W., Bauer A. Q., Stewart F. R. (2012). Bidirectional relationship between functional connectivity and amyloid-β deposition in mouse brain. *Journal of Neuroscience*.

[33] Sheng J. G., Price D. L., Koliatsos V. E. (2002). Disruption of corticocortical connections ameliorates amyloid burden in terminal fields in a transgenic model of Aβ amyloidosis. *Journal of Neuroscience*.

[34] Buxbaum J. D., Thinakaran G., Koliatsos V. (2007). Alzheimer amyloid protein precursor in the rat hippocampus: transport and processing through the perforant path. *Journal of Neuroscience*.

[35] Koo E. H., Sisodia S. S., Archer D. R. (1990). Precursor of amyloid protein in Alzheimer disease undergoes fast anterograde axonal transport. *Proceedings of the National Academy of Sciences of the United States of America*.

[36] Sisodia S. S., Koo E. H., Hoffman P. N., Perry G., Price D. L. (1993). Identification and transport of full-length amyloid precursor proteins in rat peripheral nervous system. *Journal of Neuroscience*.

[37] Lazarov O., Lee M., Peterson D. A., Sisodia S. S. (2002). Evidence that synaptically released β-amyloid accumulates as extracellular deposits in the hippocampus of transgenic mice. *Journal of Neuroscience*.

[38] Harris J. A., Devidze N., Verret L. (2010). Transsynaptic progression of amyloid-β-induced neuronal dysfunction within the entorhinal-hippocampal network. *Neuron*.

[39] Yetman M. J., Lillehaug S., Bjaalie J. G., Leergaard T. B., Jankowsky J. L. (2016). Transgene expression in the Nop-tTA driver line is not inherently restricted to the entorhinal cortex. *Brain Structure and Function*.

[40] Rönnbäck A., Zhu S., Dillner K. (2011). Progressive neuropathology and cognitive decline in a single Arctic APP transgenic mouse model. *Neurobiology of Aging*.

[41] Rönnbäck A., Sagelius H., Bergstedt K. D. (2012). Amyloid neuropathology in the single Arctic APP transgenic model affects interconnected brain regions. *Neurobiology of Aging*.

[42] George S., Rönnbäck A., Gouras G. K. (2014). Lesion of the subiculum reduces the spread of amyloid beta pathology to interconnected brain regions in a mouse model of Alzheimer’s disease. *Acta Neuropathologica Communications*.

[43] Walsh D. M., Selkoe D. J. (2016). A critical appraisal of the pathogenic protein spread hypothesis of neurodegeneration. *Nature Reviews Neuroscience*.

[44] Meyer-Luehmann M., Coomaraswamy J., Bolmont T. (2006). Exogenous induction of cerebral β-amyloidogenesis is governed by agent and host. *Science*.

[45] Ridley R. M., Baker H. F., Windle C. P., Cummings R. M. (2006). Very long term studies of the seeding of β-amyloidosis in primates. *Journal of Neural Transmission*.

[46] Eisele Y. S., Bolmont T., Heikenwalder M. (2009). Induction of cerebral β-amyloidosis: intracerebral versus systemic Aβ inoculation. *Proceedings of the National Academy of Sciences of the United States of America*.

[47] Stohr J., Watts J. C., Mensinger Z. L. (2012). Purified and synthetic Alzheimer’s amyloid beta (Aβ) prions. *Proceedings of the National Academy of Sciences of the United States of America*.

[48] Stohr J., Condello C., Watts J. C. (2014). Distinct synthetic Aβ prion strains producing different amyloid deposits in bigenic mice. *Proceedings of the National Academy of Sciences of the United States of America*.

[49] Watts J. C., Condello C., Stohr J. (2014). Serial propagation of distinct strains of Aβ prions from Alzheimer’s disease patients. *Proceedings of the National Academy of Sciences of the United States of America*.

[50] Li S., Hong S., Shepardson N. E., Walsh D. M., Shankar G. M., Selkoe D. (2009). Soluble oligomers of amyloid β protein facilitate hippocampal long-term depression by disrupting neuronal glutamate uptake. *Neuron*.

[51] Fogel H., Frere S., Segev O. (2014). APP homodimers transduce an amyloid-β-mediated increase in release probability at excitatory synapses. *Cell Reports*.

[52] Wei W., Nguyen L. N., Kessels H. W., Hagiwara H., Sisodia S., Malinow R. (2009). Amyloid beta from axons and dendrites reduces local spine number and plasticity. *Nature Neuroscience*.

[53] Deboer S. R., Dolios G., Wang R., Sisodia S. S. (2014). Differential release of β-amyloid from dendrite- versus axon-targeted APP.

[54] Bakker A., Krauss G. L., Albert M. S. (2012). Reduction of hippocampal hyperactivity improves cognition in amnestic mild cognitive impairment. *Neuron*.

